# Developing Key Performance Indicators for Emergency Department of Teaching Hospitals: A Mixed Fuzzy Delphi and Nominal Group Technique Approach

**DOI:** 10.21315/mjms2022.29.2.11

**Published:** 2022-04-21

**Authors:** Rahman NIK HISAMUDDIN, Tuan Kamauzaman TUAN HAIRULNIZAM

**Affiliations:** 1 Department of Emergency Medicine, School of Medical Sciences, Universiti Sains Malaysia, Kelantan, Malaysia; 2 Hospital Universiti Sains Malaysia, Kelantan, Malaysia

**Keywords:** quality, key performance indicators, benchmarking, emergency department, academic, teaching hospital

## Abstract

**Background:**

This article demonstrates combination of the fuzzy Delphi method (FDM) and the nominal group technique (NGT) to consolidate consensus agreement within a panel of experts regarding key performance indicators (KPIs) development for emergency department (ED).

**Methods:**

Twenty-four participants for NGT and 10 experts for FDM were randomly chosen from the emergency medicine (EM) department staff list obtained from the human resource department of study centres. A set of item constructs related to KPIs was developed from the NGT session and used for the FDM session in the second phase of the study.

**Results:**

We found that 16 out of 22 and 11 out of 15 items satisfied the first prerequisite ‘*d*’ value ≤ 0.2. Ten items (45%) from the service KPI domain and six items (40%) from the academic KPI domain had expert consensus of more than 75%. A total of 16 out of 22 (73%) and 14 out of 15 (93%) fit the criteria of an average fuzzy number (*A* value) of more than 0.5. Fifteen items that fulfilled the prerequisites were retained for the final KPI draft.

**Conclusion:**

The FDM and NGT analyses reached experts’ consensus on the suitability of the pre-selected items in the ED KPIs. The development of the KPI framework is expected to enhance future improvement of EM services and academic activities in all teaching hospitals in the country.

## Introduction

Considering the importance of the academic emergency department (ED) in the healthcare and education system, and the high mortality and morbidity rate of patients referred to the ED, it is crucial to provide quality services not only to the public but also to create an optimum and conducive atmosphere for teaching and learning activities for medical trainees (1). A set up of quality assurance programme is required to assist the service and teaching providers to continuously monitor the department performance. Ideally, each institution should create standardised and systematic data collection that is measurable, such as key performance indicators (KPIs), which serve mainly as continuous quality management. The KPIs can also be used as a benchmarking process for other well-established centres (2–3). KPIs are the elements of an organisational plan that express what it wants to achieve at a certain period.

Currently, there is no standardised and common KPI framework created for emergency medicine (EM) specialty in teaching hospitals in Malaysia. KPIs of an organisation provide an obvious commitment to improving the quality of teaching and learning, safety, and quality of patient care, ensuring safety surveillance and continuous activities for reducing dangers that threaten patients and staff (4). Therefore, given the vital role as well as the perpetual and indispensable service provided by the ED, it is necessary to re-evaluate the manner of service provision and teaching activities in these departments according to acceptable standards and criteria so that observance of these criteria will lead to improvement of EM in teaching hospitals in the country. Hence, this study was an effort to create an acceptable framework of KPIs both for service and academic activities for EM teaching hospitals in the country.

We employed a nominal group technique (NGT) and fuzzy Delphi method (FDM) to reach expert consensus on KPIs for teaching hospitals. The combination of the two techniques has not been used anywhere else in the past (5). The qualitative and qualitative data collection involved specialists and experts in the field of EM who were working in all teaching centres that run the EM trainee programme at the time of the study. The development of this standardised KPI framework is expected to enhance future improvement of EM services and academic activities in all teaching hospitals in Malaysia.

## Methods

This was a cross-sectional study that utilised mixed methods qualitative and quantitative methods NGT and FDM to obtain consensus from experts on the service and academic KPIs of teaching hospitals (6–7). The principal investigator acted as the main facilitator who provided the expert with online Google form-based questionnaires. He also collected answers to these questionnaires and comments. The facilitator then filtered out irrelevant information. This process avoided groupthink and the problems associated with group dynamics. The facilitator created, validated and performed reliability testing on the questionnaires and sent them to the participants via WhatsApp and Telegram telephone text messaging platforms. The study involved two phases.

### Phase 1 (Nominal Group Technique)

The principal investigator listed all potential initial draft KPIs to be assessed by the chosen participants. In this study, experts were considered experienced in ED employment of more than 5 years, either as clinical specialists, nursing staff, paramedics or administrators in EM, and involved in scholarly activities in the specialty of EM. For this reason, we used a purposive sampling approach for the participants, based on the lists of EM staff employed in teaching hospitals obtained from the administrative offices of the study centres. At the time of this study, there were three major teaching hospitals in the country that provided a specialty training programme in EM for the country. The selection of NGT members reflected the population that intended to use the KPIs. The list of items was decided based on the literature and the investigator’s opinion.

The NGT was conducted to create and reach agreement on the initial pool or draft of KPI. The NGT involved 24 participants, comprising equal numbers of emergency physicians (*n* = 6), senior EM residents (*n* = 6), senior ED nursing staff (*n* = 6), and senior ambulance paramedics (*n* = 6). The experts were divided into three domain discussion groups of equal numbers of participants (*n* = 5 per group) that covered three areas: i) structure; ii) process and iii) outcome (SPO), as suggested by the Donabedian model. Each group consisted of eight experts and was led by a moderator who was a senior consultant and lecturer in EM. Structure describes the context in which care and teaching activities are delivered, including hospital buildings, staff, financing and equipment. Process denotes the transactions between patients and providers throughout the delivery of healthcare and academic activities. Outcomes refer to the effects of healthcare on the health status of patients and populations and the effects of teaching activities, such as students graduating on time and scientific publications. A total of six domains were discussed and developed, consisting of the SPO for each service and academic components. The lead investigator instructed the moderators to guide the discussion on KPI development for both the service and academic activity provision of teaching hospitals. Each group was allocated a scribe from the clerical staff of the EM department. The scribes were tasked with documenting and recording all the pertinent KPI points and parameters agreed upon by the experts in each group.

### Phase 2

Phase 2 involved a different group of experts from the EM department, who were randomly selected by the principal investigator to fit the same criteria for NGT. FDM was used to obtain expert consensus on the feasibility and ranking of the top five prioritised KPIs that were agreed upon by experts from the NGT session for final use in the EM department of teaching hospitals. A set of KPI assessments was formed using a five-point Likert scale (Table 1).

The prerequisites for reaching an expert consensus consisted of three elements: i) each item in the domain achieve a threshold value (*d*) ≤ 0.2; ii) each item within the construct achieve an expert consensus of more than 75% (8) and iii) to rank the items within the constructs by calculating the average fuzzy numbers (*A* value). Items were accepted if the *A* value was more than 0.5 (9). The number of fuzzy scales must be selected in odd numbers, such as 3, 5, 7 and 9. Higher fuzzy scale value indicated that the data obtained was more accurate. The survey was distributed to the experts in Google form formats via WhatsApp or the Telegram text messaging platform.

### Fuzzy Delphi Method Implementation Steps

#### Step 1: Selection of experts

In the selection of experts from a homogenous group of experts, good results can be obtained even with small panels of 10–15 individual (10). In this study, the investigator chose 10 experts within the field of EM. The criteria for selection and random selection were similar to those of the NGT phase.

#### Step 2

**Figure 1:**
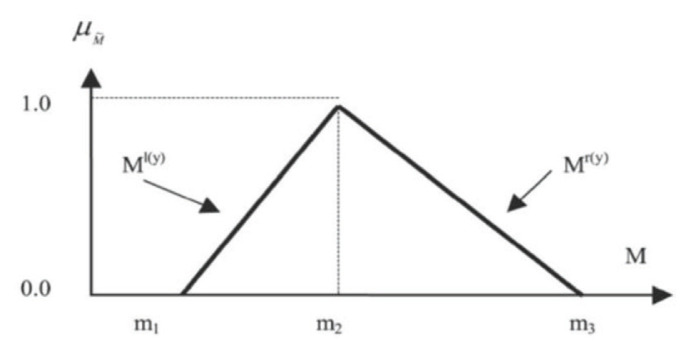
The triangular fuzzy numbers

The next step involves the conversion of all linguistic variables into triangular fuzzy numbers. A triangular fuzzy number represents the value of *m1*, *m2*, *m3*, and is symbolised as (*m1*, *m2*, *m3*). The *m1* value indicates the minimum value, the *m2* value indicates a reasonable value and the *m3* value indicates the maximum value. Figure 1 shows the values of *m1*, *m2*, and *m3* for the triangular fuzzy number. The *m* values represented the percentage likelihood that the experts agreed that the KPIs were important (i.e. for Likert scale 3: *m1* = a minimum of 20% of the experts agree it was important; *m2* = reasonably average likely 40% of the experts agree it was important; *m3* = at most 60% of expert agree it was important).

#### Step 3

**Figure 2:**
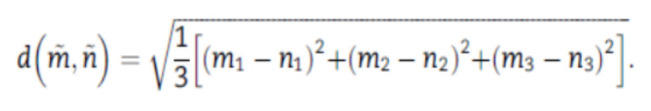
Formula for determining the distance between two fuzzy numbers

The following step was used to identify the value of threshold ‘*d*’. The threshold value is very important in identifying the level of agreement among experts. To obtain expert agreement for each item, the threshold value must not exceed 0.2 (11). A *d* < 0.2 indicates that all the experts reached an agreement on the item; otherwise, the second round would proceed to survey whether the item is needed or not. This was the first requirement criterion that needed to be fulfilled for the FDM (Figure 2).

To obtain the threshold value ‘*d*’, the following formula was used:

#### Step 4

The second criterion of requirement for the FDM involved determining the extent of the expert’s agreement, whether it was greater than or equal to 75% for each item. If the percentage of expert agreement was greater than or equal to 75% agreement for each item, then the item was assumed to reach the expert agreement. The percentage of expert’s agreement was calculated by using the formula:


$$\frac{\text{Numbers of item }d<0.2\times 100\%}{\text{Total items}}$$

#### Step 5

As the third criterion of requirement for the FDM, an α-cut greater or equal to 0.5 indicated that the item was acceptable, as it showed the consensus of experts to receive the item (12).

#### Step 6

In this step, ranking or sub-phasing of the items was performed. The ranking step involved selecting the item based on the defuzzification value (value *A* as above) based on the experts’ agreement, where the highest value of the item was determined by the most important ranking in the model.

The data entry from the Likert scale obtained was translated into fuzzy number data and analysed using the FDM programme in Microsoft Excel software. This data analysis technique is known as the fuzzy Delphi or FDM technique. The study was approved by the host institution and conducted in accordance with the Declaration of Helsinki on ethical principles regarding human experimentation developed for the medical community by the World Medical Association (WMA).

## Results

A total of 24 and 15 experts were chosen for each NGT and FDM phases, respectively. A total of 37 KPI drafts were identified by participants at the end of the NGT session for all six domains for each service and academic KPI component (22 and 15 KPIs for service and academic activities, respectively) (Tables 2 and 3).

A 100% response rate was obtained from all 10 experts for the FDM phase. All the items within the six domains had an average Likert score of 3–5, which was on the scale of moderately appropriate to extremely appropriate. These scores were converted into fuzzy numbers. Post-FDM analysis for the service and academic KPI, 16 out of 22 items and 11 out of 15 items satisfied the first prerequisite *d* ≤ 0.2. For the second prerequisite, 10 (45%) items from the service KPI domain and 6 (40%) items from the academic KPI domain had expert consensus of more than 75%; these included about 43% of the total items in the questionnaire. For the third prerequisite, 16 out of 22 (73%) items and 14 out of 15 (93%) items fit the criteria of an average fuzzy number (*A* value) of more than 0.5. We discarded 22 (59%) items and the remaining items that fulfilled the prerequisites were retained for the final draft of the content validation process. Apart from discarding items based on these prerequisites, a slight modification of items in terms of the structure, position and wording was done based on the comments from the experts. These minor changes did not alter the objective and nature of the items.

**Figure 3:**
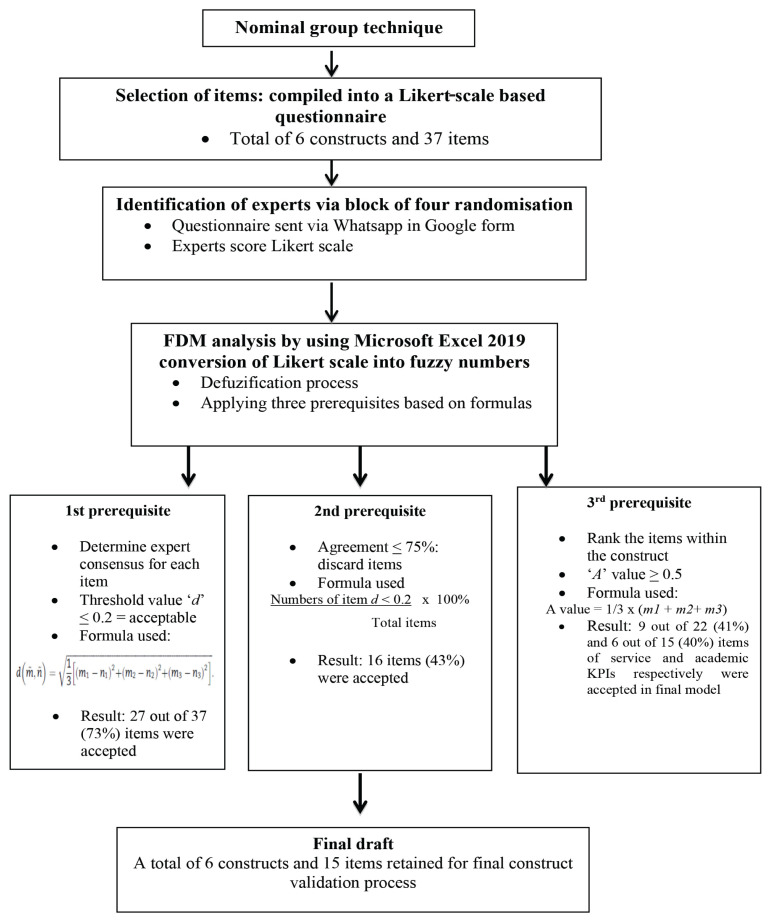
Summary of content validation using the FDM

The final model of FDM indicated that a total of 9 out of 22 (41%) items and 6 out of 15 (40%) items of service and academic KPIs, respectively, were accepted by expert consensus based on the three prerequisites. The findings are summarised in Tables 4 and 5, and Figure 3.

## Discussion

This study introduces FDM’s potential application in obtaining expert’s opinions and consensus on a decision. This method can be used as a tool to select suitable items or a content validation process before subjecting it to a construct validation process (13). Most importantly, this method provides a proper quantitative approach to usual group discussions or meetings, which are qualitative. The KPI framework can be considered a prototype that is established and consented to by experts without any prejudice, and it can be used for the targeted population after confirmatory or construct validation processes.

Phase 1 of the study utilised NGT to design and develop the initial draft framework of the KPI, which covers both the service and academic components of the department. Thirty-seven initial drafts of KPIs were developed based on ED users’ consensus within the department. Senior emergency physicians and nursing staff who were selected randomly throughout the country further analysed the applicability and appropriateness of the selected KPIs using a Likert scale; the selected KPIs were then analysed by using FDM. FDM has the advantage of being able to rank the importance of selected items and remove unfit items based on expert consensus; hence, it can serve as a content validation process (14). However, post-FDM analysis, only 15 items fulfilled all the prerequisites. About 59% of the items did not match the terms; thus, those items were regarded as failing to achieve consensus from the expert panel and were removed. These unfit items were the fuzziness or uncertainty among the expert panels that were not detected by the usual Likert scale scoring system. Each expert had his/her own uncertainty toward a certain variable, which is often regarded as the ‘grey area’. FDM is used to deal with those ‘gray areas,’ ensuring a qualified analysis outcome. Furthermore, this method catered to all the experts’ opinions, considering that some experts were more experienced, some were more knowledgeable, some had relevant skills, and some had policymaking authority in the field (15).

A teaching or university hospital serves not only as a service to the public but also as a site where teaching, learning, and research activities are carried out (16–17). Academic activities in this setting are commonly intermingled with service provision because clinical areas are the actual laboratory utilised by teachers and students for knowledge transfer and skill gain. Therefore, it has always been a tremendous challenge for the ‘frontliners’ and academicians in the ED of a teaching hospital to deliver services and academic activities without compromising quality dimension expectations of timeliness, efficiency, effectiveness, equity, safety and patient or student centredness (18). Hence, it is crucial for any ED of a teaching hospital to establish quality indicators and programmes to ensure the sustainability of quality care and academic activities that benefit both patients and students. The quality indicators can be used to evaluate ED performance. It can be utilised as a benchmarking process for all public and teaching hospitals, specifically as a measure of the success of the ED’s work process (19). Setting up KPIs for an organisation and department needs careful planning. Wrong KPI selection will defeat the purpose of KPI setup, burden resources and have a negative impact on the organisation. Hence, this study developed a set of KPIs for an ED of a teaching hospital by utilising two phases of a scientific approach: i) NGT and ii) FDM (20, 22). The KPI framework was set up using expert consensus in the field of service and the academic field of EM.

At present, medical researchers rarely use FDM to establish expert consensus on any subject matter. We recommend that FDM should be widely used in medical-related studies to get expert’s opinions and consensus, especially in developing a protocol, module or guidelines related to medical practices (23–24). Even though limited, there are studies that utilised this method for healthcare related studies. In particular, FDM is well suited to the research needed to inform health education and health promotion campaigns, set up guidelines or choose clinical management based on expert opinion (25).

## Strengths and Limitations

The method developed in this study can be used as a pre-construct validation tool to select suitable items before subjecting them to a construct validation process. Most importantly, this method provides a proper quantitative approach to usual group discussions or meetings, which are qualitative in nature. The developed questionnaire can be considered acceptable by the experts without any prejudice, and it can be used for the targeted population after the confirmatory validation process. This method will certainly reduce the risk of bias by ensuring anonymity and welcoming the opinion or atypical views among the experts and the responses are completely independent without the fear of being judged by other individuals usually present in any routine group discussions or meetings. However, one of the weaknesses of this method includes the need to constantly remind the experts to give their responses. This might lead to emotional bias among the experts. Further, the KPI framework established in this study might not be applicable to other settings elsewhere. Different organisations may have other priorities in KPI development that are more suited to their needs. The developed KPI has not been tested in real clinical and academic settings; hence, it can be considered as a prototype. Further analysis is required for its applicability in a real setting before any improvements can be made.

## Conclusion

Post-FDM analysis, the experts’ consensus on the suitability of the pre-selected items on the KPI questionnaire set was obtained. The framework is now ready for further construct validation processes and tests for its applicability in the real service and academic setting.

## Figures and Tables

**Table 1:** Level of agreements and fuzzy scale (5 points)

Linguistic variables	Likert scale	Fuzzy scale
Not appropriate at all	1	(0.0, 0.0, 0.2)
Minimally appropriate	2	(0.0, 0.2, 0.4)
Moderately appropriate	3	(0.2, 0.4, 0.6)
Very appropriate	4	(0.4, 0.6, 0.8)
Extremely appropriate	5	(0.6, 0.8, 1.0)

**Table 2:** NGT outcomes for service KPI

Domain/Items	KPI item description (Service structure-SS)
SS-1	BLS/ACLS/ATLS/PALS certification for all medical doctors working in ED (KPI outcome 80% of all doctors per any one certification)
SS-2	Minimum nursing to bed ratio in red zone (KPI: target 1:2)
SS-3	Minimum doctors to bed ratio in red zone (KPI: target 1:3)
SS-4	Maximum duration ambulances downtime annually (KPI: twice breakdown per ambulance per year)
SS-5	Annual budget allocation for point of care test (KPI: adequate to fulfill all tests request)
SS-6	Amount of Personal Protective Equipment provided and supplied annually (KPI: adequate to fulfil the use requirement)

	**KPI item description (Service processes-SP)**

SP-1	Door to time to be seen by doctors/nurses in critical (red) zone (KPI: 0 min)
SP-2	Door to time to be seen by doctors/nurses in semi-critical (yellow) zone (KPI: maximum 30 min)
SP-3	Door to time to be seen by doctors/nurses in non-critical (green) zone (KPI: maximum 120 min)
SP-4	Door to CT scan for CVA patient (KPI: within 30 min of arrival)
SP-5	Door to needle for thrombolysis in CVA (KPI: within 90 min of arrival)
SP-6	Door to thrombolytics for AMI (KPI: within 30 min of arrival)
SP-7	Ambulance response time (KPI: 15 min from call received at dispatch centre for hospital-based ambulance services)
SP-8	Number of working hours per week for medical officers (KPI: maximum 70 h per week)
SP-9	Number of working hours per week for nurses (KPI: maximum 60 h per week)
SP-10	Hand hygiene practice among staff (KPI: 100% compliance)

	**KPI item description (Service outcome-SO)**

SO-1	Percentage of success thrombolysis in AMI (KPI: 70% of all cases thrombolysed)
SO-2	Percentage of success thrombolysis in CVA (KPI: 70% of all cases thrombolysed)
SO-3	Staff happiness index (KPI: 80% of staff is satisfied working in the department at any time)
SO-4	Number of patient/public complaints (KPI: maximum five complaints annually)
SO-5	Incidence of needle prick injury in department (KPI: 0 incidence annually)
SO-6	Incidence of nosocomial infection among staff (KPI: 0 incidence)

**Table 3:** NGT outcomes for academic KPI

Domain/Items	KPI item description (Academic structure-AS)
AS-1	Ratio lecturer to trainee in EM (KPI: 1:4)
AS-2	Ratio lecturer to undergraduate in EM (KPI: 1:2)
AS-3	Ratio lecturer to housemen in EM (KPI: 1:2)
AS-4	Percentage of lecturers in ED hold research grant at any time (KPI: minimum 10%)
AS-5	Percentage of lecturers in ED with subspecialty certification (KPI: minimum 10%)

	**KPI item description (Academic process-AP)**

AP-1	Annual continuing medical education (CME) hours among the postgraduate MMed Emergency Medicine (KPI: minimum 50 h annually)
AP-2	Annual CME hours among the undergraduate (KPI: minimum 20 h per rotation)
AP-3	Annual CME hours among the housemen/junior residence (KPI: minimum 20 h per rotation)
AP-4	Teaching facilities and infrastructure downtime i.e. IT, lecture halls, manikin simulation) (KPI: Maximum two times breakdown annually)

	**KPI item description (Academic outcome-AO)**

AO-1	Number of publications produced by lecturer annually (KPI: minimum two papers annually)
AO-2	Percentage of postgraduate EM qualify at stipulated time (KPI: minimum 75% of all candidates)
AO-3	Percentage of housemen pass the rotation at stipulated time (KPI: minimum 75% of all housemen)
AO-4	Percentage of postgraduate EM able to complete dissertation at stipulated time (KPI: minimum 75% of all candidates)
AO-5	Percentage of postgraduate students (MMed) satisfied with the residency programme (KPI: minimum 80% of all candidates)
AO-6	Percentage of undergraduate students (MD) satisfied with rotation programme in ED (KPI: minimum 80% of all students)

**Table 4:** Summary of all three prerequisite post-fuzzy Delphi analysis findings for service KPI domain

Domain/Items	Average Likert score	Threshold value *d* < 0.3	Percentage of expert consensus	Average of fuzzy numbers (*A* value)	Ranking	Verdict***
Service structure (SS)						
SS-1	4.7	0.147	90	0.740	2	Retained
SS-2	4.0	0.360	80	0.613	4	Discarded
SS-3	3.9	0.343	30	0.587	5	Discarded
SS-4	3.2	0.267	60	0.447	6	Discarded
SS-5	4.4	0.147	100	0.680	3	Retained
SS-6	4.8	0.098	100	0.760	1	Retained
Service process (SP)						
SP-1	4.3	0.257	70	0.660	4	Discarded
SP-2	4.3	0.196	80	0.640	5	Retained
SP-3	3.6	0.387	30	0.527	8	Discarded
SP-4	3.9	0.344	30	0.593	6	Discarded
SP-5	4.5	0.214	90	0.700	3	Retained
SP-6	4.9	0.055	100	0.780	1	Retained
SP-7	3.1	0.225	70	0.427	10	Discarded
SP-8	3.3	0.370	50	0.473	9	Discarded
SP-9	3.9	0.227	60	0.587	7	Discarded
SP-10	4.8	0.098	100	0.760	2	Retained
Service outcome (SO)						
SO-1	3.1	0.220	70	0.420	5	Discarded
SO-2	3.2	0.208	70	0.440	4	Discarded
SO-3	3.8	0.313	50	0.567	3	Discarded
SO-4	1.9	0.213	70	0.207	6	Discarded
SO-5	4.4	0.251	90	0.687	1	Retained
SO-6	4.3	0.252	90	0.667	2	Retained

Note:

*** Prerequisite for retaining items based on expert consensus:
Threshold value *d* < 3.0 Percentage expert agreement ≥ 75% Average fuzzy value (*A* value) ≥ 0.5

All three must be satisfied to retain the items

**Table 5:** Summary of all three prerequisite post-fuzzy Delphi analysis findings for academic KPI domain

Domain/Items	Average Likert score	Threshold value *d* ≤ 0.3	Percentage of expert consensus	Average of fuzzy numbers (*A* value)	Ranking	Verdict***
Academic structure (AS)						
AS-1	3.6	0.269	60	0.520	2	Discarded
AS-2	3.4	0.367	30	0.480	5	Discarded
AS-3	3.5	0.275	60	0.500	4	Discarded
AS-4	3.6	0.302	60	0.527	1	Discarded
AS-5	3.5	0.333	50	0.507	3	Discarded
Academic process (AP)						
AP-1	4.1	0.110	90	0.620	1	Retained
AP-2	3.6	0.269	60	0.520	4	Discarded
AP-3	3.8	0.159	80	0.560	2	Retained
AP-4	3.6	0.265	70	0.527	3	Discarded
Academic outcome (AO)						
AO-1	4.0	0.306	20	0.600	5	Discarded
AO-2	4.4	0.147	100	0.680	1	Retained
AO-3	4.1	0.165	80	0.620	3	Retained
AO-4	4.4	0.183	90	0.680	1	Retained
AO-5	4.0	0.244	40	0.607	4	Discarded
AO-6	4.3	0.214	90	0.660	2	Retained

Notes:

*** Prerequisite for retaining items based on expert consensus:
Threshold value *d* < 3.0 Percentage expert agreement ≥ 75% Average fuzzy value (*A* value) ≥ 0.5

All three must be satisfied to retain the items
